# Whole health and chiropractic: a commentary on historical roots, contemporary relevance, and future directions

**DOI:** 10.1186/s12998-026-00676-6

**Published:** 2026-09-02

**Authors:** Michele Maiers, Krista Ward, Michael Ramcharan, Logan Benjamin, Wren Burton, Zachary Cupler, Maruti Ram Gudavalli, Stephanie Halloran, Benjamin Holmes, Steven Huybrecht, Jeremy Jenkins, Claire D. Johnson, Nathaniel C. Majoris, Alex Margrave, Elizabeth Moos, Vanessa Morales, Ryan D. Muller, Steven Passmore, Morgan R. Price, Stacie Salsbury, Kent J. Stuber, Stephanie G. B. Sullivan, Alexander R. Sundin, Maithy Ta, Qian Zhang

**Affiliations:** 1 Center for Research and Innovation, Northwestern Health Sciences University, Bloomington, MN USA; 2 RAND Research Across Complementary and Integrative Health Institutions (REACH) Center, Santa Monica, CA USA; 3 Life Chiropractic College West, Hayward, CA USA; 4 Southern California University of Health Sciences, Whittier, CA USA; 5 Dartmouth Health, Lebanon, NH USA; 6 Osher Center for Integrative Health, Harvard Medical School & Brigham and Women’s Hospital, Boston, MA USA; 7 Physical Medicine and Rehabilitative Services, Butler VA Health Care System, Butler, PA USA; 8 Keiser University College of Chiropractic Medicine-WPB, West Palm Beach, FL USA; 9 VA Boston Healthcare System, Boston, MA USA; 10 Department of Orthopaedic Surgery, Duke University School of Medicine, Durham, NC USA; 11 VA Pacific Islands Healthcare System, Honolulu, HI USA; 12 Texas Chiropractic College, Pasadena, TX USA; 13 National University of Health Sciences, Lombard, IL USA; 14 VA Connecticut Healthcare System, West Haven, CT USA; 15 Palmer College of Chiropractic, Davenport, IA USA; 16 Parker University, Dallas, TX USA; 17 VA Iowa City Health Care System, Iowa City, IA USA; 18 Kinesiology and Recreation Management, University of Manitoba, Winnipeg, CA Canada; 19 VA Puget Sound Health Care System, Seattle, WA USA; 20 Palmer College of Chiropractic, Davenport, IA USA; 21 Canadian Memorial Chiropractic College, Toronto, ON, CA Canada; 22 Life University, Marietta, GA USA; 23 Northwestern Health Sciences University, Bloomington, MN USA; 24 KC Care Health Center, Kansas City, MO USA; 25 Port Orange, FL USA

**Keywords:** Chiropractic, Health promotion, Holistic health, Patient-centered care, Therapeutic alliance

## Abstract

**Background:**

This commentary describes perspectives from workshop attendees at a North American educational conference on the chiropractic profession and its relationship with whole health. Whole Health is a paradigm shift within healthcare settings and funding agencies in the United States that utilizes a systems framework to transform healthcare. Whole health frameworks emphasize biopsychosocial well-being as defined by individuals, families, and communities, and leverages interprofessional, team-based approaches to promote well-being, disease prevention, and restoration of health.

**Main body:**

Two separate, 60-minute workshop sessions were convened at the 2025 Association of Chiropractic Colleges Education Conference and Research Agenda Conference to discuss whole health and chiropractic. Attendees (*n* = 105) included chiropractic academicians, researchers, practitioners, students, and organizational leaders from institutions largely based in the United States. Participants in 14 small groups discussed (1) ways the chiropractic profession aligns with whole health, and (2) how chiropractors can contribute to whole health in clinical practice and healthcare education. Notes taken by group scribes were collected from each workshop and generated 401 distinct comments. Thematic analysis with an inductive approach was used to group and define key topics that emerged from the data. Aspects of the chiropractic profession were described as historically being aligned with whole health for its foundational philosophy in innate healing and resilience, and for espousing person-centeredness and patient values. Themes describing the future contribution of chiropractic to whole health include evidence-based practice; communication and advocacy; integration into the broader healthcare system; and forward-facing education. There was overlap between past and future when considering holism; therapeutic alliance and patient empowerment for self-care; direct access; therapeutic conservatism; preventive health and lifestyle medicine; and longitudinal care.

**Conclusions:**

The whole health paradigm presents an opportunity for chiropractors to be recognized contributors within multidisciplinary teams, using their expertise to aid chronic disease prevention, patient empowerment, and health care delivery innovations. Continued education and training, policy changes, and further research are required for the chiropractic profession to best serve patients and provide value within a whole health framework.

**Supplementary Information:**

The online version contains supplementary material available at https://doi.org/10.1186/s12998-026-00676-6.

## Background

There is a growing movement, particularly in the United States, to reform healthcare towards a whole health, wellness model [1, 2]. Whole health emphasizes person-centric care, well-being alongside disease prevention, and multisectoral collaboration in healthcare delivery. A recent report by the National Academies of Sciences, Engineering and Medicine in Washington, D.C. defines whole health as “physical, behavioral, spiritual, and socioeconomic well-being as defined by individuals, families, and communities. It leverages interprofessional, team-based approaches to care, anchored in longitudinal relationships, to promote well-being, disease prevention, and restoration of health” [3].

Both whole health and the chiropractic profession trace their origins to a desire to reform or redesign healthcare, and address limitations in prevailing healthcare systems [4–7]. Whole health represents a departure from historically mechanistic, disease-oriented medical care, toward a framework that promotes well-being and fosters self-efficacy. Further, it broadens the biopsychosocial model and emphasizes relationships between providers, patients, and communities. Aspects of chiropractic’s historical roots align closely with this view.

Chiropractic care, since its inception in the midwestern United States during the late 19th century, has been grounded in a vitalistic, holistic view of health [8, 9]. The public generally tends to associate chiropractic with care of the spine, including treatment of back and neck pain, and a holistic style of healthcare [10]. Historically, chiropractic philosophers credited the vitalistic healing power of nature, advancing the theory that health is the natural state and that the innate tendency of the body is to seek and maintain homeostasis [11–13]. As such, salutogenesis, or health creation, is considered an outcome of care [12]. While some chiropractors have focused solely on spinal manipulation or the correction of subluxation as the means for achieving salutogenesis [14], early hiropractic also embodied holism, a non-reductionist view of health including the well-being of the mind, body, and spirit. Holism was a view necessitating consideration of the entirety of an individual as opposed to treating only a condition or symptoms, and addressing the underlying factors contributing to a patient’s condition within their life’s context [15]. Chiropractic has focused on pain, disability, and understanding spine-related causes of disease. In some sectors, this has centered on the elucidation and treatment of the vertebral subluxation–a concept, like vitalism and holism, which has both provided a focus for and caused division within the profession [16, 17]. While these tenets within the chiropractic profession have evolved alongside decades of research and changing societal values, there has been a recent convergence of these historical principles with contemporary healthcare in the context of a whole health paradigm [18–21].

Contemporary chiropractic care fits squarely within the whole health paradigm by recognizing the role of personal goals, patient preferences and interpersonal relationships [22, 23]; public health [9]; and lifestyle medicine [24, 25]. A recent US practice analysis suggests that most chiropractors incorporate health promotion strategies into their practice, addressing lifestyle factors and disease prevention alongside treating conditions [26]. Furthermore, the discipline’s growing collaboration with other healthcare providers underscores its role in delivering team-oriented care [26], especially for individuals who require long-term clinical management and self-care for chronic musculoskeletal conditions [27]. 

This commentary aims to highlight and further define the role of chiropractic care within the whole health paradigm. Drawing on findings from a professional workshop conducted within the U.S. and informed by current best practices, research, chiropractic education, and various care delivery and policy initiatives, we propose that chiropractic is well-positioned to contribute to whole health initiatives at the individual, healthcare system, and population level. This role builds on chiropractic’s publicly recognized role as experts in spinal pain and disability to amplify parallel efforts in health promotion, lifestyle medicine, and supporting the well-being of individuals, families, and their communities [10].

## Approach

The information presented in this commentary is a synthesis of de-identified comments shared by workshop participants at the 2025 Association of Chiropractic Colleges Education Conference and Research Agenda Conference (ACCRAC) and related literature acquired by the authorship team. This commentary is not intended to produce generalizable knowledge, represent the chiropractic profession at large, nor the institutions that employ members of the authorship team. Rather, the intention was to explore both past alignment and future opportunities for whole health within the chiropractic profession.

### Workshop summary

Two separate 60-minute workshop sessions with different cohorts of attendees were convened and conducted at the Association of Chiropractic Colleges Education and Research Agenda Conference, ACCRAC, held in New Orleans, Louisiana, USA, during March of 2025 [28]. Attendees included a cross section of academicians, researchers, practitioners, students, and organizational leaders, largely from the chiropractic profession and living in North America, primarily but not exclusively in the U.S. Contextual background information about whole health was presented, including definitions of whole health from the U.S. Department of Health and Human Services National Institutes of Health, and the National Academies of Sciences, Engineering and Medicine in Washington, D.C. (Table 1).

**Table 1:** Definitions related to whole health

Organization and Source	Definition
*National Center for Complementary and Integrative Health. NCCIH Strategic Plan FY 2021–2025: Mapping the Pathway to Research on Whole Person Health [* 29 *]*	Whole person health involves looking at the whole person—not just separate organs or body systems—and considering multiple factors that promote either health or disease.
*National Academies of Sciences Achieving Whole Health: A New Approach for Veterans and the Nation [* 3 *]*	Whole health is physical, behavioral, spiritual, and socioeconomic well-being as defined by individuals, families, and communities. Whole health care is an interprofessional, team-based approach anchored in trusted relationships to promote well-being, prevent disease, and restore health.

1. Workshop participants gathered in small groups to discuss the following: Identify the ways that the chiropractic profession aligns with whole health.
2. Discuss how chiropractors can contribute to whole health in clinical practice and healthcare education.

A note taker for each small group documented key discussion points, and a reporter shared these perspectives with the larger group. The discussion was facilitated and key messages were projected in real-time to acknowledge responses and maintain a visual guide for the discussion. Notes taken by small group scribes were collected at the end of each workshop. Attendees were invited to indicate their interest in (1) acknowledgment and (2) participating as a contributing author for this paper.

Insights from each small group were entered into a spreadsheet for analysis. As the intersection of chiropractic and whole health has not previously been reported in the literature, an inductive approach to thematic analysis was used to organize and categorize participant insights, grouping and defining key topics that emerged from the data [29]. Analysis was performed independently by two authors (MM, KW), who used a sorting method within the spreadsheet to organize, define, and identify relationships among the findings. They met twice to reconcile their findings. A third author (MR) reviewed the findings for face validity with the original output from the workshops. Direct quotes from workshop participants are in quotation marks; contextual narrative and in-text citations reflect contributions by the co-authors. Participants who expressed an interest in co-authoring this commentary were invited post-workshop to provide additional material via email, including (1) background resources and evidence-supported perspective on either whole health or chiropractic that connects the profession historically with whole health principles and (2) share their thoughts and related sources contextualizing chiropractic in whole health relative to the broader healthcare system and current and anticipated public health needs. The first author (MM) incorporated co-authors’ contributions into the thematic findings from the workshop.

A total of 105 attendees participated in either of two 60-minute workshop sessions, 94 of whom provided their names at the end of the workshop to be acknowledged in the paper. Seventy-six workshop participants expressed interest in co-authoring the paper; 25 met the requirement for authorship by submitting post-workshop material and contributing to writing and revision. The first three authors (MM, KW, MR) synthesized the workshop notes, co-author feedback, and related literature through multiple rounds of revision.

This project is not intended to produce generalizable knowledge. The information and opinions shared by participants are presented here in aggregate, based on deidentified notes from the workshop. As such, the project was determined not to be human subjects research by the Life Chiropractic College West Institutional Review Board. Our intention to publish this commentary was verbally communicated to participants at the beginning of each session. Attendees were informed that content generated during the workshops could be used to develop this manuscript, and that their contributions may be included in the paper.

## Themes

**Fig. 1:**
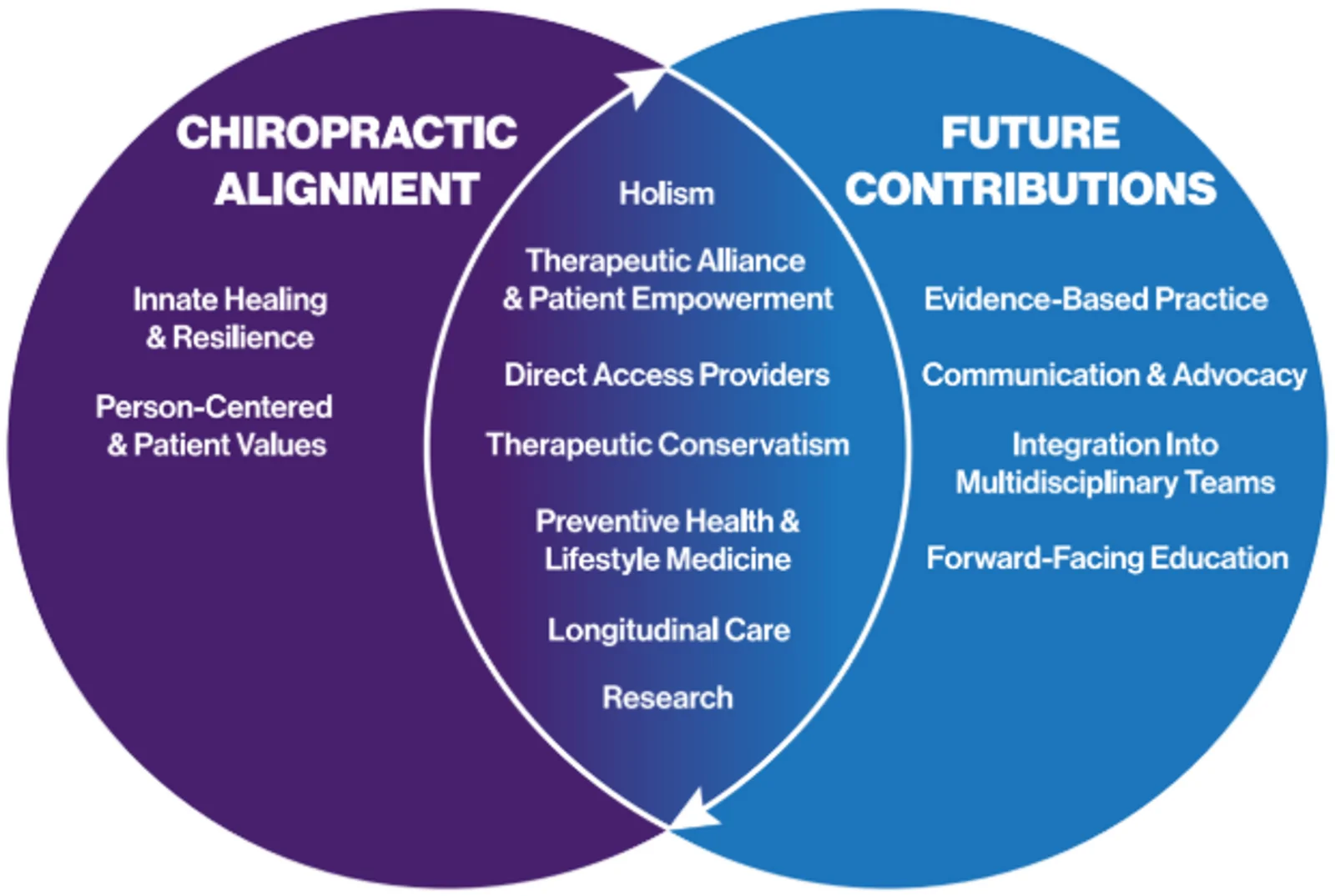
Historical alignment, future contributions, and their intersection for chiropractic and whole health

Written notes from 14 small groups generated 401 distinct comments, subsequently grouped into 14 themes organized with consideration to which question they were in response to, as well as how they fit contextually. There was considerable overlap of themes generated between the 2 questions, and many were bi-directional in that they pertained to both historical alignment of the chiropractic profession with whole health and the future role of chiropractic clinical practice and education on whole health (Fig. 1).

### Historical alignment of chiropractic with whole health

#### Innate healing and resilience

The earliest definition of chiropractic states that “The functions are performed by Innate in health and disease” [30]. This vitalistic philosophy emphasizes the body’s resilience, and its natural ability to heal. Parallels to this were drawn by workshop participants to whole health, which encourages resilience and adaptability among individuals and communities by activating self-care and employing lifestyle medicine to support innate health [31]. As one group of workshop participants summarized, “Health is not merely the absence of sickness, but the presence of flourishing and vitality.”

#### Person-centered and patient values

Early in chiropractic’s history, the founder of chiropractic defined its purpose “…so that physical and spiritual health, happiness and the full fruition of earthly life may be fully enjoyed” [30]. A more contemporary way of phrasing this is advocated by the U.S. Veterans Health Affairs’ Whole Health Program, which asks patients “what matters to them” in their health care seeking journey [32]. Chiropractic and other complementary and integrative therapies have historically attracted people seeking alternatives to conventional healthcare that better align with their personal values and cultural preferences [33].

Workshop participants described chiropractors as having a “patient-centered focus,” “meeting patients where they are at,” and “advancing human potential from the patient’s perspective.” They also noted that the “tenets of chiropractic care are empathy and physical touch” and that chiropractors’ “multi-modal scope of practice” with a “wide range of treatment options” allows for choice in patient care. Patient-centered care was identified as fundamental to healthcare quality, as affirmed by the Institute of Medicine (now the National Academy of Medicine) as one of the six aims for improvement [34].

### Themes overlapping between chiropractic alignment and future contributions

#### Holism

It has been noted that chiropractic has historically espoused the need to assess the “whole” patient from a structural, emotional, chemical, and nutritional perspective [15, 18]. Workshop participants made multiple notes about what is now commonly described as a biopsychosocial approach to care. They highlighted how chiropractors are educated to consider more than the chief complaint and deliver care that addresses both the presenting issue as well as good health generally [35, 36]. Holistic care is a concept that continues to evolve alongside developments in research, practice, and cultural expectations.

Particularly salient to the public health aspect of whole health is the need for chiropractors to become more conversant about how health is influenced by the context of where people live, work, study and play [37]. It was asserted that chiropractors should screen for socioeconomic risk factors and, as participants noted, “know community resources to execute collaborative care”. One author put forward the example that chiropractors can document socioeconomic health risk factors with ICD-10 Z-Codes that allow healthcare providers to identify and address various socioeconomic barriers to health [38].

#### Therapeutic alliance and patient empowerment

Therapeutic alliance refers to the collaborative relationship between a patient and their healthcare provider. This is characterized by mutual respect, shared decision-making, and a joint commitment to achieving desired health outcomes [39]. With an historical foundation of trusted relationships and high patient satisfaction [40], chiropractors have a unique opportunity to leverage therapeutic alliance to provide education about the causes and prevention of a range of chronic diseases and connect patients to community resources. Chiropractors can also empower patients with self-care for lifestyle and behavioral change through coaching and goal setting to encourage individuals to take an active role in their health and make informed decisions to improve long-term well-being [41, 42]. As one small group suggested, chiropractic needs to lean into a paradigm shift away from “you need me” toward “you’ve got this”.

#### Direct access providers

In the U.S., the National Center for Health Workforce Analysis projects a shortage of 87,150 full-time equivalent (FTE) primary care physicians by 2037, which will be especially acute in non-metropolitan areas [43]. In the face of these shortages, chiropractors can provide guideline concordant care as direct access providers within the healthcare system. This may be particularly important in rural and medically underserved areas [44, 45] where seeking referrals to specialists can present both financial and geographic barriers to care. Chiropractic as a profession includes a diversity of practices addressing a range of community healthcare needs, filling roles across settings that range from primary contact/conventional, to specialist/complementary. Efforts to decrease persistent disparities in utilization of chiropractic care across socioeconomic groups present clear opportunities for professional growth and future contributions [46, 47].

As direct access providers, chiropractors are often sought out for care related to low back, neck, and other musculoskeletal pain relief [48]. However, chiropractic visits also offer checkpoints that can detect at-risk patterns before they escalate into multi-site chronic conditions [49]. Chiropractors commonly provide lifestyle advice; formalizing this with best practices in public health prevention would enhance chiropractors’ role in whole health. For example, individuals experiencing pain are at increased risk of self-harm [50]. One author explained that, given disparities in access to mental health and pain specialists, chiropractors acting as direct-access providers serve as an accessible frontline for biopsychosocial-informed care. Multiple workshop groups also noted that there is a significant opportunity for chiropractors to “recognize and triage mental health.” Chiropractors can engage in primary, secondary, and tertiary suicide prevention efforts to identify those at risk of self-harm and connect individuals with public health resources or behavioral health treatment [51, 52].

#### Therapeutic conservatism

The profession’s historical tenets of therapeutic conservatism mirrors today’s stepped care approach of delivering therapeutic interventions [53, 54]. This philosophy contends that care is best initiated using the least invasive treatment options prior to care escalation [55] and is reflected in best practice guidelines for conditions commonly treated by chiropractors, including pain generally and back pain specifically [56]. Offering evidence-based, guideline-concordant first-line care options for most spinal pain complaints can improve management of this pervasive pain condition and reduce care escalation and downstream costs for patients [57–62], enhancing what many participants referred to as “value-based care.” Prioritizing non-invasive interventions and gradually escalating care only when warranted reflects a whole health philosophy.

#### Preventive health and lifestyle medicine

As health systems evolve to emphasize prevention and interdisciplinary collaboration, chiropractic offers a scalable, evidence-aligned approach to expanding access to whole health care [60, 63–65]. Workshop participants described how the chiropractic profession aligns with whole health through its emphasis on spine care, preventative care, movement/function, and healthy lifestyle (e.g. sleep, physical activity, nutrition, ergonomics). Participants also described the need for the profession to “educate on health behaviors,” and “model positive lifestyle activities.”

Over 40 years ago, the World Health Organization stated: “The role of the health sector must move increasingly in a health promotion direction, beyond its responsibility for providing clinical and curative services” [66]. Chiropractic management has a role in addressing primary, secondary, and tertiary prevention of many health conditions [67]. Recent practice analysis research shows that chiropractors frequently provide health promotion services including counseling on physical activity, diet, and stress reduction, reflecting their commitment to whole health principles [26, 65]. Despite this, preventive health screening and lifestyle medicine are rarely covered as a reimbursable service when delivered by a chiropractor [68], and therefore the occurrence of this service may not be documented during the clinical encounter.

#### Longitudinal care

Traditionally, most chiropractors delivered care in private practice, independent and outside of conventional healthcare systems. This holds true today, although to a lesser extent as more integrated opportunities for practice have been created [26]. Participants identified one perceived benefit of the private practice business model is that it enables the development of long-term relationships with patients, caring for entire families and over generations [69]. Working in practices so closely integrated within communities provides unique opportunities to influence community health and foster environments that support sustained, longitudinal lifestyle change. One group described this as “influencing generational health and mindsets”.

One reported barrier to practicing whole health across disciplines is the perceived increase in provider – patient interaction time [70]. Chiropractic treatment plans commonly include multiple visits over a course of care, and care is frequently reinitiated due to the chronic episodic nature of conditions like spinal pain [26, 71]. While per-visit time varies [72], increased visit frequency may provide additional opportunities for chiropractors to develop therapeutic alliances, observe health longitudinally, and support whole health habits. One workshop small group summarized this as “multiple touchpoints for multiple aspects of health.”

#### Research

Workshop participants called for “high-quality research to drive the profession forward,” and to “contribute to the research community on the values of whole health and chiropractic care.” Early writings by D. D. Palmer sought to understand the elements, and interconnectedness, of what is now known as whole health: “Great truths…are the result of consecutive investigation…What are the properties by which organs, or the organism as a whole, are maintained? Upon what does the performance of functions depend? What is metabolism and what relation does it bear toward vitality?.To possess this knowledge will add much to our welfare and happiness…” [30]. Participants cited the potential for chiropractic researchers, academic institutions and practice-based research networks to investigate Palmer’s musings with contemporary research. Examples include developing measures of whole health, investigating the value of whole health to communities served by chiropractors, and identifying effective dissemination and implementation strategies to integrate whole health practice behaviors.

### Future contributions through clinical practice and education

#### Evidence-based practice

Many workshop participants stressed the ongoing need for chiropractors to incorporate research findings on whole health into practice. Examples included adding quality of life and well-being indicators to standardize collected outcome measures and incorporating U.S. Preventive Task Force Guidelines into clinical care [73]. Clinical Compass, formerly The Council on Chiropractic Guidelines and Practice Parameters, recommends evidence-based clinical methods that combine scientific evidence with clinical judgment and patient preferences, including patient education for self-management [74]. Clinical practice guidelines for musculoskeletal pain management also highlight the important role chiropractors can play in delivering health promotion and preventive services. This includes (1) screening for risk factors for disease, (2) evidence-based health behavior counseling to promote health, and (3) manual procedures, including spinal manipulation, to enhance patients’ ability to engage in active lifestyles and engage in self-care and exercise [56, 67, 75]. The WHO released its first guideline on non-surgical management of chronic primary low back pain in 2023 [76]. It provides recommendations for interventions “delivered in primary and community care settings” and is intended for multidisciplinary clinicians, including chiropractors. Care must consider “physical, psychological, and social” factors and tailor treatment to the individual. The guideline advocates for holistic, person-centered management emphasizing a biopsychosocial framework and the need for integrated, coordinated care that reflects patients’ individual contexts and determinants of health.

The shift toward whole health is anticipated to expedite high-quality, guideline-concordant care, reduce medical costs and waste, and remove some of the current stressors placed on an overwhelmed healthcare system [77]. These assumptions are currently being studied with funding from the National Institute of Health in the Implementation of the American College of Physicians Guideline for Low Back Pain trial [78]. Studies on environments actively applying clinical practice guideline driven assessment and care delivery can bridge the evidence to practice model. The successes and challenges of guideline implementation should be considered alongside alignment with whole health [79].

#### Communication and advocacy

Workshop participants expressed the need for chiropractors to “become expert communicators” at patient-provider, inter-provider, and profession-community levels. From “using motivational interviewing” with patients to “communicating well with other disciplines” and “educating national-level health organizations about chiropractic’s role,” participants emphasized the importance of communication, with emphasis on the need to embrace a stronger advocacy role. By actively representing chiropractic within healthcare organizations, public forums, and policy conversations, chiropractors can more effectively practice, promote, and advance whole health principles.

Chiropractors’ contribution to preventive health services requires expanding their role in the healthcare system beyond a focus on spinal and musculoskeletal conditions, to include broader participation in evidence-based preventive health delivery pathways and payment structures that reflect these services [67, 80]. This expanded contribution depends on “building trust,” “better organization/less in-fighting,” and “stakeholder and policy maker engagement.” By advocating for equitable reimbursement models, integration in public-health initiatives, and recognition of chiropractic’s role in prevention and wellness, the profession can strengthen its position in shaping healthcare policy and community health outcomes.

#### Integration into multidisciplinary teams

Workshop participants often noted that chiropractic’s integration into multidisciplinary care settings is accelerating [81, 82], particularly as the profession contributes to pressing public health needs like chronic pain management and opioid alternatives [67, 83–86]. There is evidence for the effectiveness of this approach in settings ranging from private practices to federally funded, hospital-based programs, like the U.S. Veterans Health Administration [84, 85, 87]. Some among these have incorporated chiropractic into whole health service lines. During the workshop, participants emphasized the need for interdisciplinary collaboration and “being a part of the multidisciplinary team– being a ‘team player’.” By understanding their role within broader healthcare systems, chiropractors become valuable team members, co-managing patients with providers from various fields, including medicine, physical therapy, and mental health [88]. This requires learning a shared healthcare language, interfacing with interoperable electronic health record systems, and aligning with population health priorities to enhance communication and collaboration. Appropriate communication and collaboration are predicated on chiropractors’ ability to articulate their value proposition for delivering high-value spine care [74, 89, 90]. Integrating chiropractic into healthcare systems may enhance patient access to high-value, cost-effective spine care, without the need for subsequent referrals. Integrating chiropractic into multi-disciplinary settings may also improve patient access, a need workshop participants noted, especially for medically underserved patients who face economic or geographic barriers to accessing primary care or specialist services [59]. Ideas included providing chiropractic care in “teaching satellite/community clinics” and working in Federally Qualified Health Centers (FQHCs), Tribal Health and employer-based clinics. There are examples of chiropractors already successfully working in these multi-disciplinary settings [91–93]. Further, it was noted that community health clinics offer unique opportunities for chiropractors to contribute and enhance existing whole health environments [94, 95].

#### Forward-facing education

The need for ongoing curriculum development that emphasizes whole health was frequently noted by workshop participants. Future chiropractic education should emphasize competencies to promote patient self-efficacy and behavior change through team-based care, shared decision-making, and effective goal-setting strategies to promote improved outcomes [96]. Whole health-oriented continuing education can enhance the skills of experienced clinicians to more effectively engage the broader healthcare system and create networks of providers who share whole health values.

Through interprofessional educational (IPE) opportunities and experiential learning, chiropractic students, residents, and doctors can engage in collaborative scholarship and practice among various healthcare disciplines. Doing so can develop critical skills essential for delivering comprehensive, patient-centered care in the modern healthcare environment. Recent scoping reviews in health professional education demonstrate that effective IPE typically includes experiential learning, community-based training, problem-based approaches, and technology-enhanced modalities, all of which contribute to improved collaborative behaviors and patient-centered care [97]. These methods have been identified as key factors positively influencing IPE outcomes and should inform future chiropractic curriculum design. Moreover, IPE initiatives consistently show gains in interprofessional communication, clarity of professional roles, and collaborative teamwork—outcomes directly supportive of whole health models.

Finally, post-graduate training such as residencies and fellowships may offer an avenue to refine advanced competencies in team-based care [62]. These training opportunities can embed chiropractors directly into whole health care teams, providing experiential immersion that supports long-term integration and professional development [98].

#### Call to action

The chiropractic profession is witnessing a pivotal moment in healthcare transformation, particularly within the United States but also worldwide. The paradigm shift required to realize whole health offers a powerful opportunity for the chiropractic profession to position itself as an integral component of the health care delivery system, rather than an alternative or peripheral option. The following calls to action are the authors’ proposed directions for chiropractic stakeholders to consider, attempting to highlight key, actionable findings built on the observations of workshop attendees and critical reflection of the themes presented in this commentary. They underscore efforts needed to meaningfully contribute to whole health with appropriate evidence, training, scope-of-practice clarity, referral pathways, interprofessional collaboration, and safeguards.

1. Recognize holism as a foundational tenet of chiropractic and evolve our understanding of this concept. This includes developing greater cultural awareness and agility; integrating mental and emotional health into patient-centered care plans; promoting self-care practices; and connecting with community resources to address socio-economic barriers to health [22].
2. Embed whole-health competencies—including psychosocial assessment, interdisciplinary collaboration, patient self-efficacy and trauma informed care—into core training within chiropractic pre- and post-graduate curricula [99, 100].
3. Hold the chiropractic profession accountable to clinical standards and the latest research in whole health and preventive healthcare practices, grounding interventions in science to maximize patient benefit.
4. Foster a culture of lifelong learning that embraces humility, innovation, and cross-disciplinary partnership with the communities serviced by chiropractors [101].
5. Pursue research focused on the whole health and preventive benefits of chiropractic encounters on musculoskeletal function, injury prevention, and chronic disease management [102].
6. Integrate chiropractic services into care pathways for spine pain, both within and adjacent to conventional healthcare settings. Interoperable electronic health record systems should be used to facilitate referrals, promote communication, and integrate across healthcare delivery, public health and social support services [103–105].
7. Reduce structural barriers to the integration of chiropractic care into a whole health system of care. This includes insurance coverage for evidence-based preventive health and lifestyle medicine when delivered by chiropractors, eliminating arbitrary limits on visits, aligning coverage policies with scope of practice, and the use of interoperable electronic health records [106].
8. Leverage longitudinal care within chiropractic offices for evidence-based preventive health screening and behavioral health interventions, and as extenders for the public and primary healthcare systems.

## Strengths

Workshop discussion was grounded in definitions of whole health provided by authoritative sources in the U.S., including the National Institutes of Health [107] and the National Academies of Sciences, Engineering, and Medicine (NASEM) [3]. Although whole health is frequently presented as a conceptual framework, it also has clearly defined operational dimensions. While workshop participants were not facilitated to focus on either conceptual or operational aspects of whole health, their discussion included both. Although no single internationally standardized model has achieved universal adoption, several authoritative frameworks provide explicit organizational structures and implementation processes. One prominent example is the NASEM framework, which characterizes Whole Health through structural principles that include person-centeredness, holistic care, and upstream prevention, as well as longitudinal, team-based processes oriented around the individual’s values, goals, and priorities [3]. These were prominent themes that emerged from the workshop and demonstrate alignment of the participants’ perspectives with recognized experts on whole health.

## Limitations

This commentary is not intended to represent the position of the entire chiropractic profession relative to a whole health paradigm. Rather, we aimed to convey the perspectives of attendees engaged in this topic at a research conference, most of whom were affiliated with academic institutions and healthcare delivery systems in North America. The workshop discussions may not mirror attitudes across the profession, and dissenting views may not have been expressed in this public setting. The discussion was largely positive toward whole health and the chiropractic profession’s position within it as the workshop prompts were designed to elicit perceived alignment and future contributions, rather than to explore non-alignment, risks, limitations, or dissenting perspectives. Questions designed to probe about negative aspects of whole health, or how chiropractic historically or presently falls short of a whole health paradigm, would have likely elicited different themes. This was outside of the scope of the workshop.

It is also possible that chiropractors from other regions of the world who work in other cultural contexts may not align with the conference group’s investment in whole health and how chiropractic meets community needs through this paradigm. The findings presented in this commentary however do align with research on North American chiropractic students [108], who express strong support for evidence-based chiropractic care that stays true to traditional chiropractic principles, as well as a desire for interprofessional collaboration and a role in mainstream healthcare. Additional research is needed to identify the relevance of the whole health paradigm to chiropractors globally.

## Conclusions

The tenets of whole health can be viewed as inherent to both the historical underpinnings and future directions of the chiropractic profession. Concerted efforts to improve upon foundational values may amplify chiropractic’s relevance within the paradigm shift toward whole health, improving access to high-value care and shaping a healthcare system that prioritizes well-being over disease.

## Supplementary Information

Below is the link to the electronic supplementary material.


Supplementary Material 1


## Data Availability

The datasets used and/or analyzed during the current study are available from the corresponding author on reasonable request.

## Declarations

### Disclaimer

The views expressed in this article are those of the authors and do not necessarily reflect the official position of their affiliations, including American Chiropractic Association, World Federation of Chiropractic, or institutions with which the authors are employed. The contents of this manuscript represent the views of the authors and do not necessarily reflect the position or policy of the US Department of Veterans Affairs or the United States Government.

## Abbreviations

ACCRAC: Association of Chiropractic Colleges Research Agenda Conference
FTE: Full time equivalent
FQHC: Federally Qualified Health Centers
ICD: International Classification of Diseases
IPE: Interprofessional Education
US: United States
